# Effectiveness of traditional Chinese Medicine Breathing-Daoyin Rehabilitation Techniques on lung function and exercise endurance in COPD: a systematic review and meta-analysis

**DOI:** 10.3389/fmed.2026.1775201

**Published:** 2026-05-04

**Authors:** Can Wang, Yingying Lu, Xinxin Liu, Yudong Wu

**Affiliations:** 1 Yantai Penglai Traditional Chinese Medicine Hospital, Yantai, China; 2 Affiliated Hospital of Shandong College of Traditional Chinese Medicine, Yantai, China; 3 Beijing University of Chinese Medicine, Beijing, China

**Keywords:** chronic obstructive pulmonary disease, exercise endurance, pulmonary function, systematic review, Traditional Chinese Medicine Breathing-Daoyin Rehabilitation Techniques

## Abstract

**Objective:**

To evaluate the effects of Traditional Chinese Medicine Breathing-Daoyin Rehabilitation Techniques (TCM-BDRT) on lung function and exercise capacity in patients with chronic obstructive pulmonary disease.

**Methods:**

We conducted a comprehensive search of Chinese and English databases up to August 2025. Primary outcome measures included pulmonary function parameters (FEV1%, FVC%, PEF%, FEV1/FVC%) and exercise endurance (6-min walk test, 6MWT). Secondary outcomes comprised Chronic Obstructive Pulmonary Disease Assessment Test (CAT) scores, blood gas variables, and incidence of adverse events. Meta-analysis was performed using Stata 17.0.

**Results:**

Eleven RCTs involving 964 participants were included. The meta-analysis demonstrated that Traditional Chinese Medicine Breathing-Daoyin Rehabilitation Techniques significantly improved FEV1% [MD = 5.00, 95% confidence interval (2.95, 7.05)], FVC% [MD = 6.34, 95% CI (4.04, 8.63)], peak expiratory flow rate percentage [MD = 6.55, 95% CI (4.93, 8.18)], and 6-min walk distance [MD = 19.53 m, 95% CI (7.94, 31.12)]. It also reduced arterial blood carbon dioxide partial pressure [MD = −5.99, 95% CI (−8.28, −3.69)] and increased arterial oxygen partial pressure [MD = 11.06, 95% CI (8.84, 13.28)]. All differences were statistically significant. No significant differences were observed in improvements in FEV1/FVC% or CAT scores. None of the included studies reported adverse events. The certainty of evidence was moderate to low.

**Conclusion:**

TCM-BDRT holds promise as a potential non-pharmacological intervention for enhancing lung function, exercise endurance, and gas exchange capacity in patients with COPD. This low-intensity, scalable therapeutic approach aligns with the integrated treatment paradigm of combining Traditional Chinese Medicine and Western Medicine. It may be particularly suitable for patients who are frail or have limited exercise capacity. Nevertheless, large-scale, multi-center, high-quality RCTs are warranted to validate its long-term efficacy, with a specific focus on safety profiles.

**Systematic review registration:**

[https://www.crd.york.ac.uk/PROSPERO/view/CRD420251114263], identifier [CRD420251114263].

## 1 Introduction

Chronic obstructive pulmonary disease (COPD) is a progressive lung disease characterized by persistent airflow limitation and chronic airway inflammation (1, 2). Global statistics indicate that COPD has become the third leading cause of death worldwide, with its prevalence projected to continue rising through 2030, placing significant strain on healthcare systems and society. Common symptoms include chronic cough, sputum production, and dyspnea. As the disease progresses, patients experience reduced exercise capacity, limitations in daily activities, and severely impaired quality of life (3). In managing this condition, the combination of bronchodilators, inhaled corticosteroids, and pulmonary rehabilitation has been proven effective in alleviating symptoms. However, it remains unable to halt the ongoing deterioration of lung function. Persistent acute exacerbations and poor long-term treatment adherence continue to be major obstacles.

In recent years, non-pharmacological therapies have gained widespread recognition as a vital component of pulmonary rehabilitation. International guidelines indicate that exercise training, as a primary evidence-based approach, effectively alleviates dyspnea symptoms and enhances physical function (4). However, traditional exercise programs often face challenges in feasibility and adherence, particularly for patients with limited physical capacity and reduced exercise endurance. TCM Breathing-Daoyin Rehabilitation Techniques (TCM-BDRT) is a traditional Chinese medicine pulmonary rehabilitation technique developed by the respiratory team of the First Affiliated Hospital of Henan University of Chinese Medicine. It is rooted in the principles of “integration of form and energy” and “combining movement with stillness,” emphasizing the coordinated unity of breathing, limb movements, and mental focus. Contemporary research indicates that respiratory training combined with Daoyin Exercise (a form of Chinese exercise therapy) can enhance respiratory muscle coordination and thoracic wall compliance, thereby improving key pulmonary function indicators (such as FEV1% and PEF%) and optimizing alveolar ventilation. These practices also reduce respiratory effort, enhance diaphragmatic endurance, and balance autonomic nervous system function, thereby alleviating dyspnea and increasing exercise tolerance (as measured by the 6-min walk test). Furthermore, studies demonstrate that these methods contribute to improved mental health and overall quality of life (5).

In TCM theory, COPD falls under the categories of “pulmonary distension,” “cough,” and “wheezing.” Its primary pathological mechanism is described as “underlying deficiency with superficial excess,” with particular emphasis on the pathological state of “dual deficiency of the lung and kidney.” The goal of TCM-BDRT is to “regulate the lung to promote qi circulation and guide the kidney to consolidate original qi,” aligning with the theoretical essence that “the lung governs qi.” This therapy possesses a dual core: consolidating the foundation and nourishing the original qi while simultaneously tonifying the lungs and kidneys to promote unimpeded qi circulation (6). This rehabilitation model, Daoyin by the principle of “intent-directed breathing,” not only addresses the common issue of exercise limitation in COPD patients but also ensures long-term sustainability, aligning with the trend of integrating traditional and Western Medicine in rehabilitation practice.

Previous randomized controlled trials (RCTs) have demonstrated that TCM-BDRT positively affects symptoms, pulmonary function, and quality of life in COPD patients. However, these studies are often constrained by factors such as small sample sizes, short follow-up periods, and inconsistent outcome measures, leading to divergent conclusions. To address this gap, this study aims to quantitatively evaluate the efficacy of TCM-BDRT in improving lung function and exercise endurance in COPD patients through a systematic review and meta-analysis. The research seeks to provide evidence-based guidance for clinical rehabilitation practice and support the development of a lung rehabilitation model integrating both Chinese and Western Medicine characteristics, thereby better aligning with the practical needs of China’s healthcare environment.

## 2 Materials and methods

This study follows the Preferred Reporting Items for Systematic Reviews and Meta-Analyses (PRISMA) guidelines and has been registered on the PROSPERO platform (Registration number: CRD420251114263).

### 2.1 Inclusion and exclusion criteria

(1) P: COPD patients who meet the GOLD diagnostic criteria (7), with no restrictions on gender, age, disease duration, or severity.
(2) I: The experimental group received standardized TCM-BDRT, either alone or alongside health education, respiratory muscle training, and western medicine. The source, specific operation steps, and implementation method of TCM-BDRT are provided in Supplementary material 1.
(3) C: The control group received standard health education, Western Medicine, and respiratory muscle training.
(4) O: Primary outcomes included pulmonary function (measured by FEV1%, FVC%, PEF%, FEV1/FVC%) and exercise endurance (measured by the 6-MWT).
(5) S: Only RCTs in English or Chinese were included.
(6) Exclusion criteria: (1) Duplicate publications; (2) Systematic reviews and meta-analysis, conference abstracts, and animal studies; (3) Studies with irrelevant research content; (4) Studies with mismatched interventions, inconsistent trial designs, missing full texts, or inability to extract primary outcome measures.

### 2.2 Strategy for conducting literature search

We searched eight databases, including PubMed, Web of Science, Embase, Cochrane Library, CNKI, WANFANG, VIP, and SinoMed, covering the period from each database’s inception to August 2025. Search terms included “chronic obstructive pulmonary disease,” “COPD,” “traditional Chinese medicine respiratory Daoyin,” “Daoyin,” and “randomized.” The search strategy employed both controlled vocabulary and free-text keywords, supplemented by manual searching. Detailed search procedures are provided in Supplementary material 2.

### 2.3 Literature screening and data extraction

Two reviewers independently reviewed the literature and collected relevant data. Any discrepancies were resolved with the assistance of a third researcher. The collected data included authors, publication year, sample size, patient characteristics, disease duration, details of the intervention, treatment duration, and outcome measures.

### 2.4 Quality assessment of studies

Two reviewers conducted quality assessments using the Cochrane Risk of Bias tool (RoB 2.0) (8). Any disagreements were resolved by consulting a third reviewer. The assessment focused on areas including random sequence generation, allocation concealment, blinding implementation, completeness of outcome data, and selective reporting. Each domain was categorized as “low risk,” “risk of bias unclear,” or “high risk.”

### 2.5 Statistical analysis

We performed statistical analyses using Stata 17.0. Continuous data are presented as mean difference (MD) and 95% confidence interval (CI). Heterogeneity was assessed using the Q test and I^2^ statistic. A fixed-effect model was applied when *p* ≥ 0.10 and *I*^2^ < 50%; otherwise, a random-effects model was used. Potential sources of heterogeneity were identified through sensitivity and subgroup analyses. Additionally, publication bias was assessed using funnel plots and Egger’s test, with a significance level set at α = 0.05.

Two reviewers independently assessed the certainty of evidence for each outcome using the GRADE system. Any disagreements were resolved through discussion with a third reviewer. Evidence was rated as “high,” “moderate,” “low,” or “very low” based on factors including risk of bias, consistency, indirectness, imprecision, and potential publication bias.

## 3 Results

### 3.1 Literature screening process and characteristics of included studies

**FIGURE 1:**
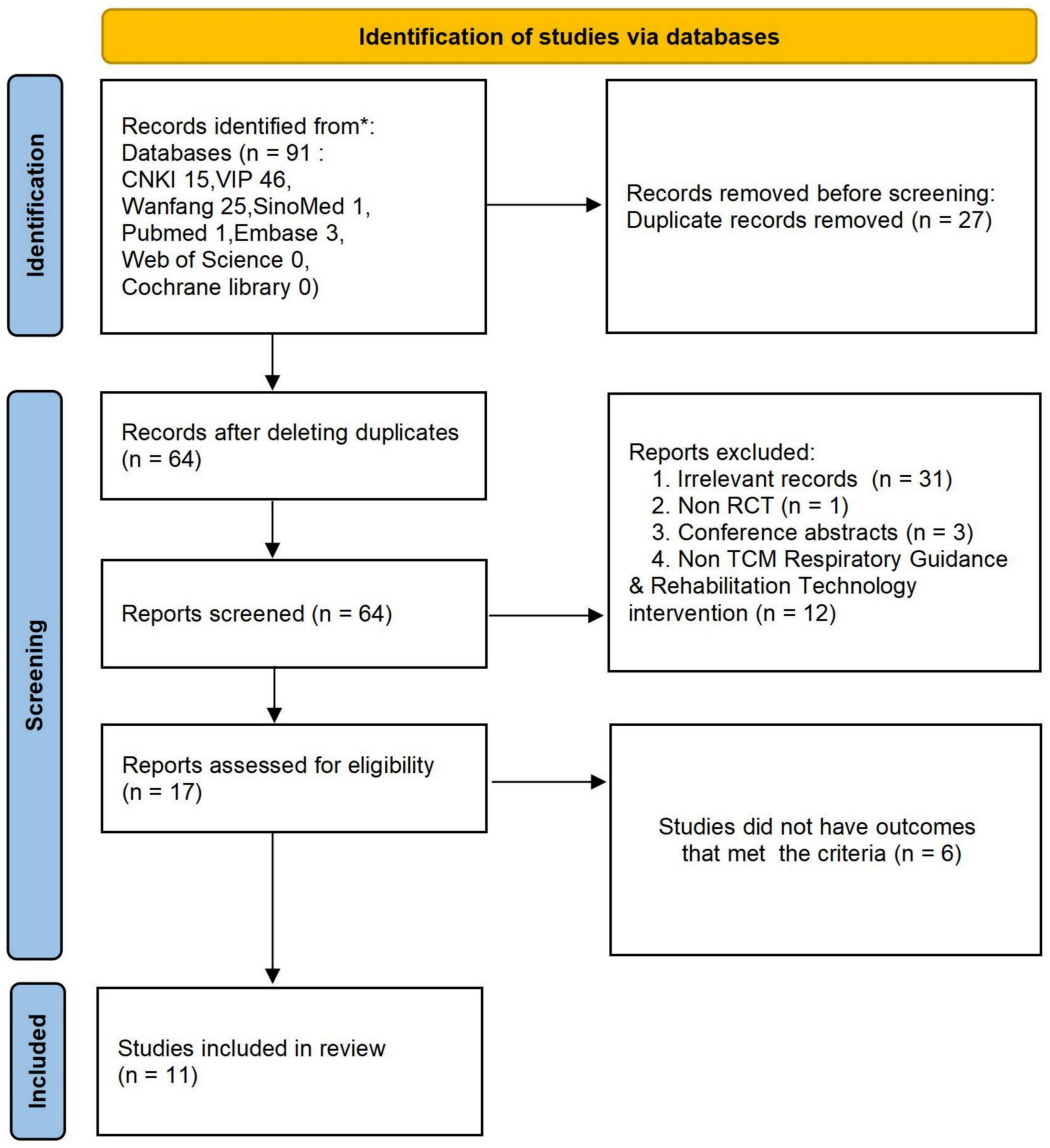
Literature screening flowchart.

A total of 91 articles were initially screened. After reviewing titles, abstracts, and full texts, 11 studies (9–19) were included, with a total of 964 patients: 481 in the combined therapy group and 483 in the control group (Figure 1 and Table 1).

**TABLE 1:** Basic characteristics of included studies.

Study ID	Grouping methods	Sample size		Gender male/ female		Age [mean (SD)]		Disease duration (a)		Intervention measures		Dose (mg)/ Treatment Cycle (days)	Outcomes
		T	C	T	C	T	C	T	C	T	C		
Chen et al. (9)	Random number table	30	30	18/12	17/13	57.60 ± 7.60	54.20 ± 6.50	10.40 ± 2.10	9.70 ± 1.60	TCM-BDRT + WM	WM (Long-acting beta agonists + sustained-release theophylline tablets)	NA/12w	➀FEV1%; ➂ PEF%; ➃ FEV1/FVC%; ➄ 6MWD; ➅CAT scores
Hu and Zhang (10)	Random number table	30	30	NA	NA	NA	NA	NA	NA	TCM-BDRT + HE	HE	bid/12w	➀ FEV1%; ➁FVC%; ➂ PEF%
Ji et al. (11)	Random	40	40	28/12	29/11	69.80 ± 8.60	69.50 ± 8.70	3∼7	3∼6	TCM-BDRT + WM	WM(Bronchodilators + expectorants + antibiotics + low-dose oxygen therapy)	bid/24w	➀ FEV1%; ➁ FVC%; ➂PEF%; ➄6MWD; ➆ PaCO_2;_ ➇ PaO_2_
Dong et al. (12)	Random number table	82	82	59/23	60/22	69.70 ± 4.20	69.30 ± 4.50	8.60 ± 2.70	8.70 ± 2.40	TCM-BDRT + WM	RC + WM(Bronchodilators + expectorants + antibiotics + low-dose oxygen therapy)	bid/3w	➀ FEV1%; ➃ FEV1/FVC%; ➄ 6MWD; ➅ CAT scores
Lian (13)	Random number table	61	61	39/22	38/23	56.40 ± 7.50	56.90 ± 7.10	6.10 ± 1.00	6.40 ± 1.20	TCM-BDRT	HE	bid/12w	➃ FEV1/FVC%
Wang (14)	Random number table	44	44	25/19	24/20	70.69 ± 2.64	71.24 ± 1.23	NA	NA	TCM-BDRT + RC	RC	bid/NA	➀ FEV1%; ➆ PaO_2_; ➇ PaCO_2_
Ma et al. (15)	Random	60	60	39/21	40/20	58.30 ± 4.80	58.90 ± 5.00	3.20 ± 0.80	3.60 ± 0.60	TCM-BDRT	WM(Bronchodilators + expectorants + antibiotics + low-dose oxygen therapy)	bid/3w	➀ FEV1%; ➁ FVC% ➂ PEF%; ➄ 6MWD; ➆ PaCO_2_; ➇PaO_2_
Wang et al. (16)	Random number table	30	30	23/7	20/10	67.94 ± 5.17	67.94 ± 5.10	10.48 ± 2.17	10.72 ± 2.34	TCM-BDRT	RMT	bid/8w	➀ FEV1%; ➄ 6MWD; ➅ CAT scores
Yin (17)	Random	41	41	21/20	22/19	57.23 ± 3.14	58.54 ± 3.21	NA	NA	TCM-BDRT	HE	qd/4w	➀ FEV1%; ➁ FVC% ➂ PEF%
Zhang (18)	Random	51	53	41/10	43/10	68.16 ± 7.18	65.62 ± 10.02	202.76 ± 165.80 m	180.15 ± 161.18 m	TCM-BDRT + HE	HE	bid/12w	➀ FEV1%; ➄ 6MWD; ➅ CAT scores; ➈ Adverse events
Zhang (19)	Random	12	12	NA	NA	65.92 ± 5.58	69.58 ± 4.06	42.65 ± 38.95 m	38.17 ± 40.58 m	TCM-BDRT + HE	HE	bid/12w	➃ FEV1/FVC%; ➄ 6MWD; ➅ CAT scores; ➈ Adverse events

TCM-BDRT, Traditional Chinese Medicine Breathing-Daoyin Rehabilitation Techniques; WM, Western Medicine; HE, Health Education; RC, Routine Care; RMT, Respiratory Muscle Training; w, week; m, month; a, year; T, Treatment group; C, Control group; ➀ FEV1%, Forced Expiratory Volume in the First Second percentage; ➁ FVC%, Forced Vital Capacity percentage; ➂ PEF%, Peak Expiratory Flow percentage; ➃ FEV1/FVC%, Percentage of forced expiratory volume in 1 s relative to forced vital capacity; ➄ 6MWD, 6-min walk distance; ➆ PaCO_2_, Arterial partial pressure of carbon dioxide; ➇ PaO2, Arterial partial pressure of oxygen.

### 3.2 Assessment of study quality

**FIGURE 2:**
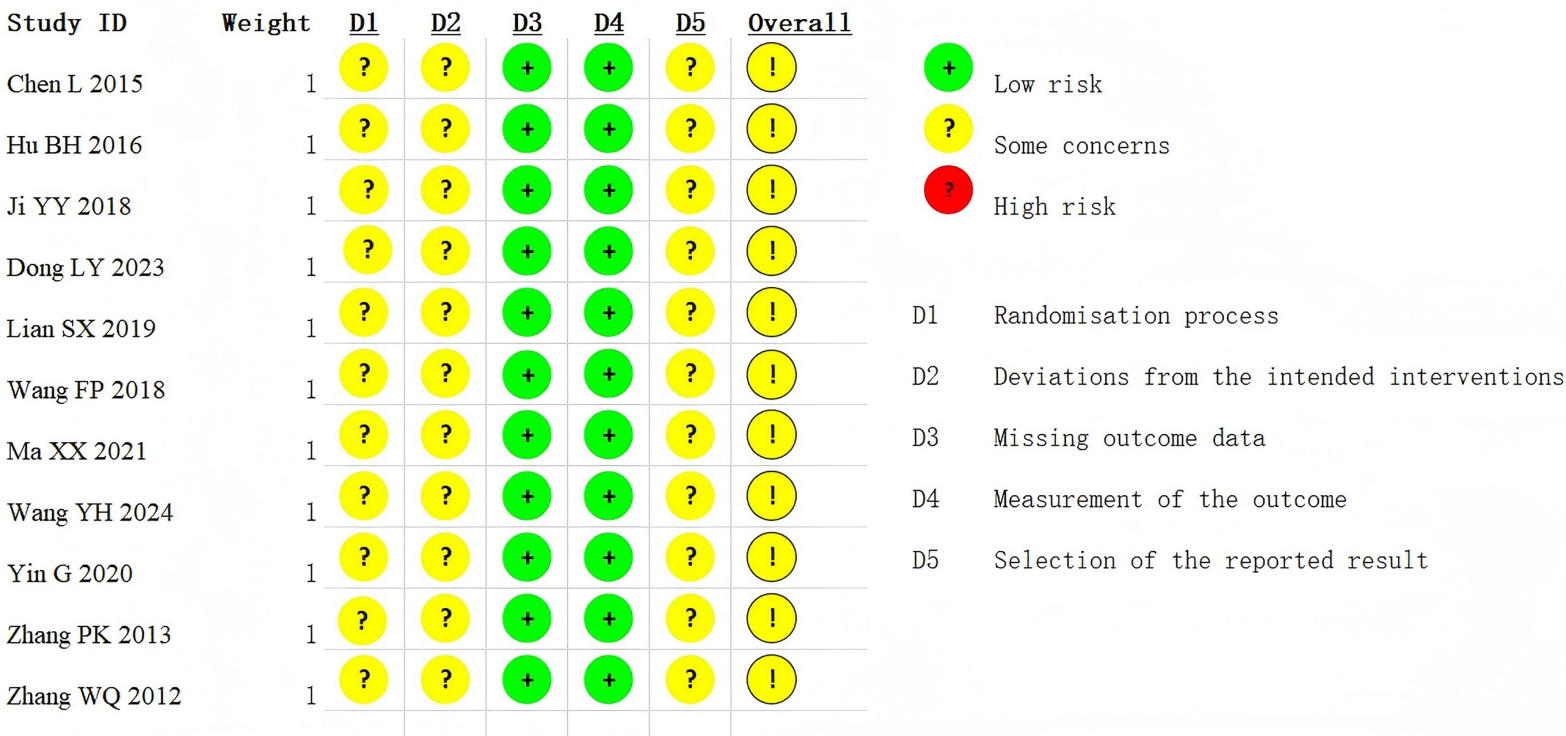
Risk of bias chart.

Among the 11 studies (9–19), 6 studies (11, 14, 15, 17–19) mentioned randomization, and 5 studies (9, 10, 12, 13, 16) provided more detailed random sequence generation. None of the studies reported the use of subject or investigator blinding. Regarding deviations from the intended interventions (D2), none of the studies reported blinding of participants or personnel, leading to a “some concerns” rating due to potential performance bias. For missing outcome data (D3), all studies reported complete outcome data or appropriately addressed dropouts, and were therefore rated as “low risk.” Concerning the measurement of outcomes (D4), all studies used standardized and objective methods, such as pulmonary function tests and the 6-min walk test, resulting in a “low risk” rating. In terms of selective reporting (D5), most studies lacked trial registration or pre-specified analysis plans, giving rise to “some concerns” for potential reporting bias. Together, the overall risk of bias across all studies was judged as “some concerns,” primarily driven by issues related to randomization, deviations from intended interventions, and selective outcome reporting (Figure 2).

### 3.3 Meta-analysis results

#### 3.3.1 FEV1%

**FIGURE 3:**
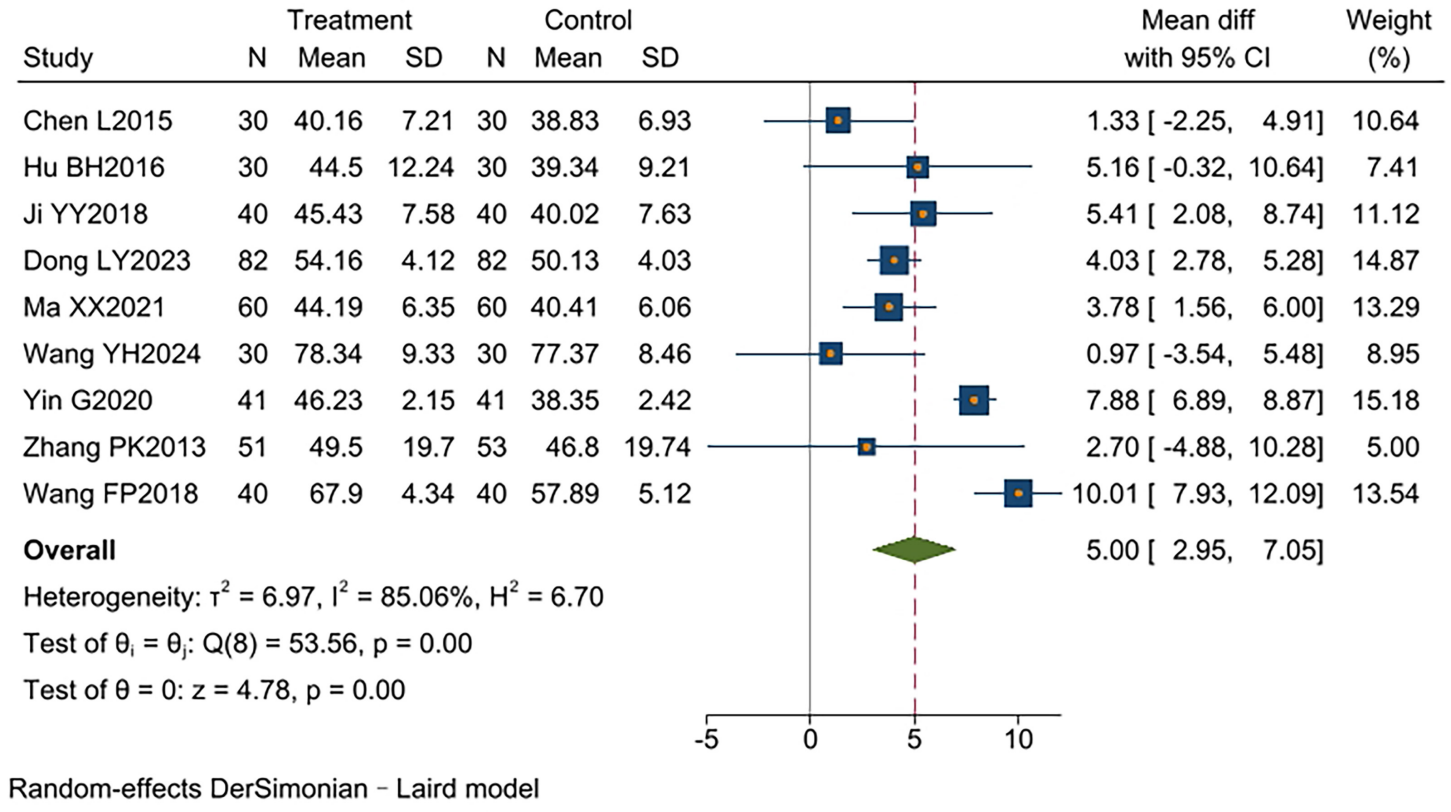
Forest plot of FEV1%.

Nine studies (9–12, 14–18) reported relevant data on FEV1%, involving a total of 810 patients (404 in the intervention group and 406 in the control group). High heterogeneity existed between studies (*I*^2^ = 85%, *p* < 0.00001). Analysis using a random-effects model showed that the experimental group had significantly higher FEV1% than the control group [MD = 5.00, 95% CI (2.95, 7.05), *p* < 0.00001]. Two studies (14, 17) did not report disease duration data, differing from the remaining studies. After excluding these two studies, heterogeneity significantly decreased (*I*^2^ = 0%, *p* < 0.00001), and the effect size became more consistent [MD = 3.78, 95% CI (2.84, 4.73), *p* < 0.00001]. The results indicated that TCM-BDRT significantly improves FEV1%, and sensitivity analysis confirms the robustness of these findings (Supplementary material 4). TCM-BDRT (Figure 3).

#### 3.3.2 FVC%

**FIGURE 4:**
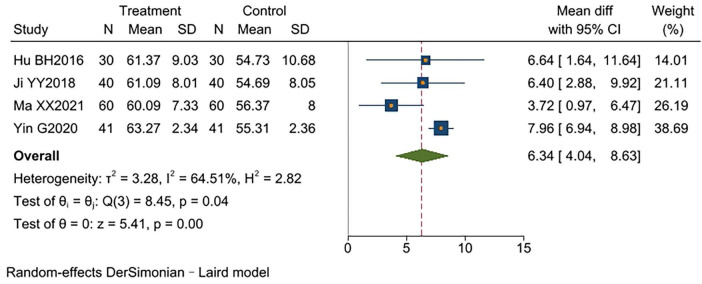
Forest plot of FVC%.

Four studies (10, 11, 15, 17) reported FVC%, involving a total of 342 patients: 171 participants in each of the experimental and control groups. These studies exhibited significant heterogeneity (*I*^2^ = 65%, *p* < 0.00001). Analysis using a random-effects model showed that the experimental group had significantly higher FVC% than the control group [MD = 6.34, 95% CI (4.04, 8.63), *p* < 0.00001]. One study (17) did not report disease duration, differing from the others. Excluding this study significantly reduced heterogeneity (*I*^2^ = 0%, *p* < 0.00001) and yielded more consistent results [MD = 5.03, 95% CI (3.05, 7.02), *p* < 0.00001]. The results indicated that TCM-BDRT significantly improves FEV1%, with sensitivity analysis confirming the robustness of these findings (Supplementary material 4). TCM-BDRT (Figure 4).

#### 3.3.3 PEF%

**FIGURE 5:**
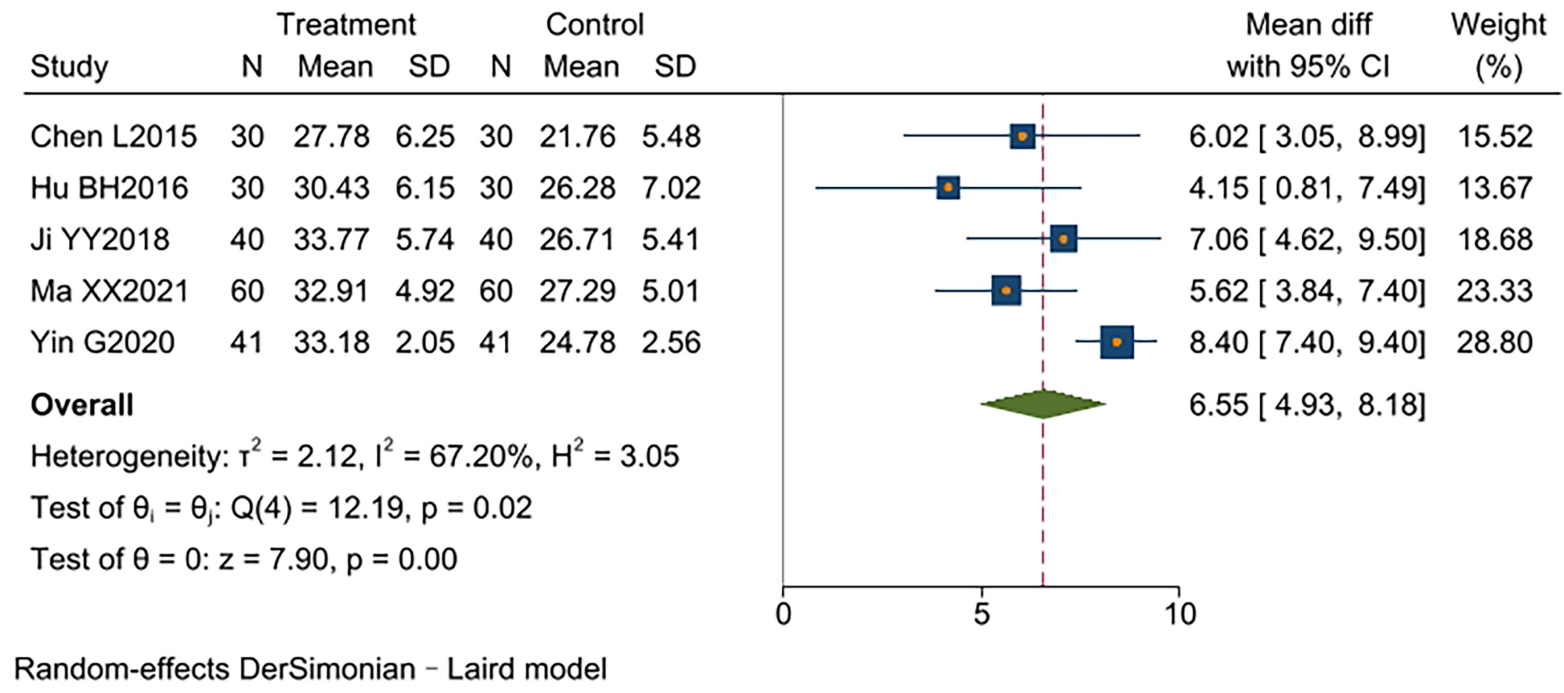
Forest plot of PEF%.

Five studies (9–11, 15, 17) reported PEF%, involving a total of 402 participants (201 in the experimental group and 201 in the control group). High heterogeneity existed between studies (*I*^2^ = 67%, *p* < 0.00001). Analysis using a random-effects model showed that the experimental group had significantly higher PEF% than the control group [MD = 6.55, 95% CI (4.93, 8.18), *p* < 0.00001]. One study (17) did not report disease duration, resulting in inconsistency with other studies. After excluding this study, heterogeneity significantly decreased (*I*^2^ = 0%, *p* < 0.00001), and results became more consistent [MD = 5.84, 95% CI (4.64, 7.05), *p* < 0.00001]. TCM-BDRT. The results indicated that TCM-BDRT significantly improves PEF% performance, with sensitivity analysis confirming the robustness of these findings (Supplementary material 4 and Figure 5).

#### 3.3.4 FEV1/FVC%

**FIGURE 6:**
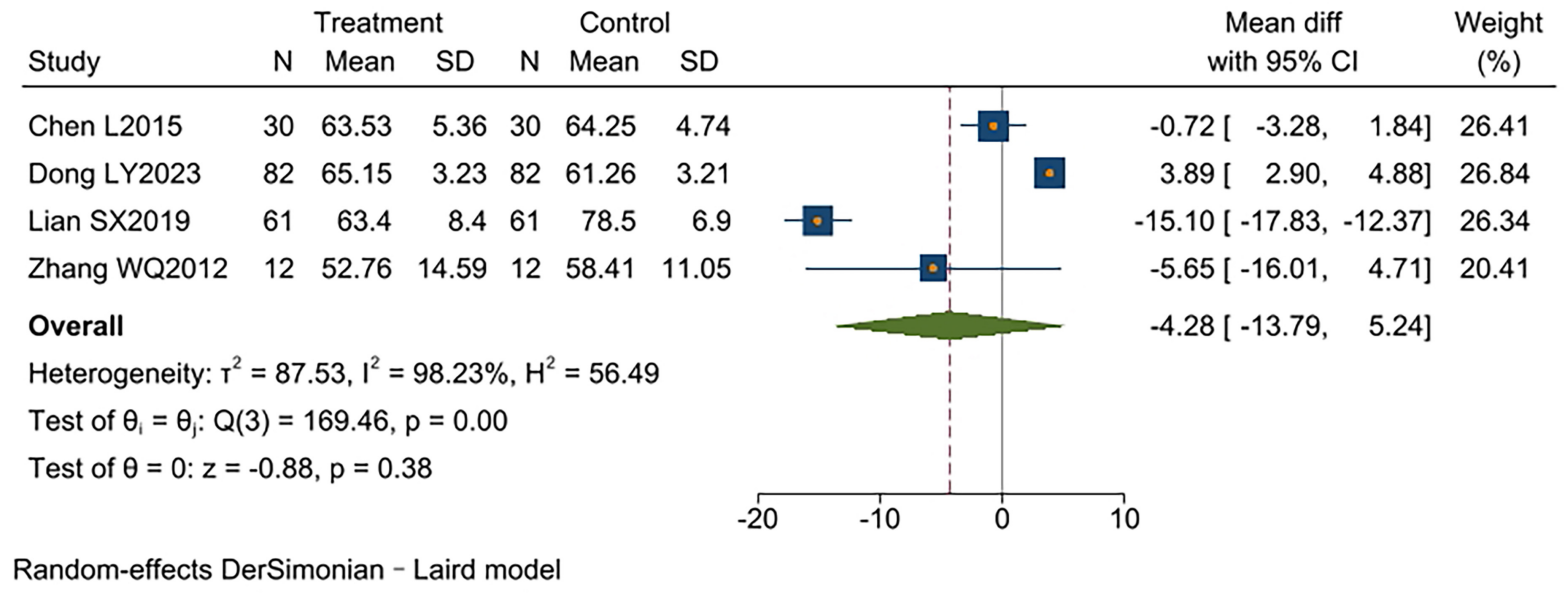
Forest plot of FEV1/FVC%.

Four studies (9, 12, 13, 19) reported FEV1/FVC%, involving a total of 370 participants (185 per group). Significant heterogeneity existed between studies (*I*^2^ = 98%, *p* < 0.00001). Analysis using a random-effects model showed no significant difference between groups [MD = −4.28, 95% CI (−13.79, 5.24), *p* = 0.38] (Figure 6).

#### 3.3.5 6 MWD

**FIGURE 7:**
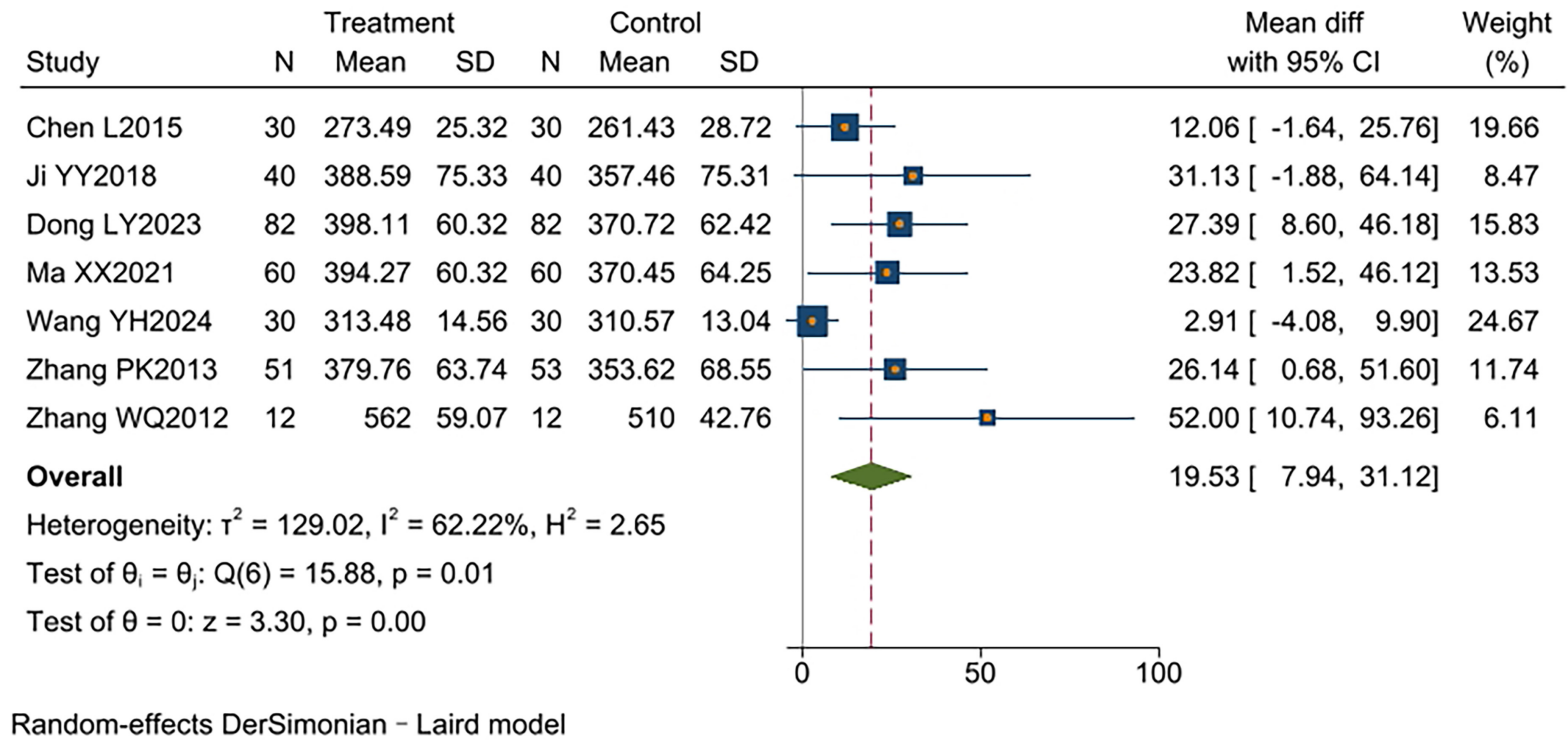
Forest plot of 6MWD.

Seven studies (9, 11, 12, 15, 16, 18, 19) reported 6MWD, involving a total of 612 participants (305 in the experimental group and 307 in the control group). Significant heterogeneity was observed (*I*^2^ = 62%, *p* < 0.00001). Random-effects model analysis showed that the experimental group had significantly higher 6MWD than the control group [MD = 19.53 m, 95% CI (10.74, 31.12), *p* < 0.00001]. Subgroup analysis showed that TCM-BDRT combined with health education significantly improved 6MWD compared with health education alone [MD = 33.77 m, 95% CI (10.65, 56.88), *p* < 0.00001]. Similarly, TCM-BDRT combined with Western Medicine significantly improved 6MWD compared with western medication alone [MD = 19.69 m, 95% CI (10.19, 29.18), *p* < 0.00001]. However, no significant improvement was observed when TCM-BDRT was compared with respiratory muscle training alone [MD = 2.91 m, 95% CI (−4.08, 9.90), *p* = 0.42] (Supplementary material 3). This demonstrates that TCM-BDRT significantly enhance patients’ exercise endurance (Figure 7).

#### 3.3.6 CAT scores

**FIGURE 8:**
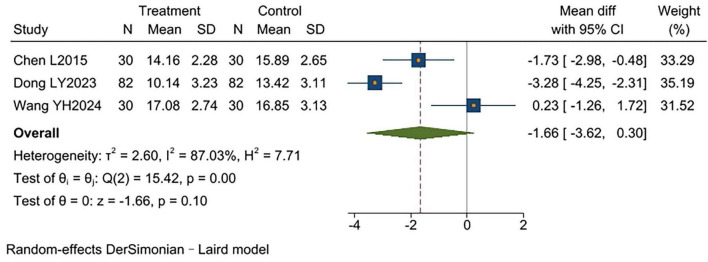
Forest plot of CAT scores.

Three studies (9, 12, 16) reported CAT scores, involving a total of 284 patients (142 in the experimental group and 142 in the control group). Significant heterogeneity existed between studies (*I*^2^ = 87%, *p* = 0.0004). Analysis using a random-effects model showed no statistically significant difference in CAT scores between groups [MD = −1.66, 95% CI (−3.62, 0.30), *p* = 0.10]. Results indicated that TCM-BDRT failed to effectively improve quality of life in COPD patients (Figure 8).

### 3.3.7 PaCO_2_

**FIGURE 9:**
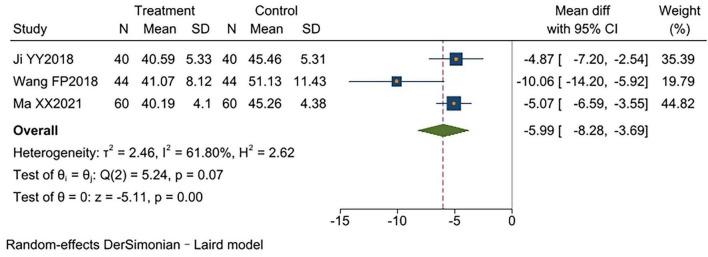
Forest plot of PaCO_2_.

Three studies (11, 14, 15) reported PaCO_2_, involving a total of 288 patients (144 per group). Significant heterogeneity existed between studies (*I*^2^ = 62%, *p* < 0.00001). Random-effects model analysis showed that the experimental group had significantly lower PaCO_2_ levels than the control group [MD = −5.99, 95% CI (−8.28, −3.69), *p* < 0.00001]. One study (14) did not report disease duration, differing from the others. Excluding this study significantly reduced heterogeneity (*I*^2^ = 0%, *p* < 0.00001). Analysis confirmed that TCM-BDRT effectively lowered patients’ PaCO_2_ levels [MD = −5.01, 95% CI (−6.28, −3.74), *p* < 0.00001], with sensitivity analysis confirming the robustness of these findings (Supplementary material 4 and Figure 9).

#### 3.3.8 PaO_2_

**FIGURE 10:**
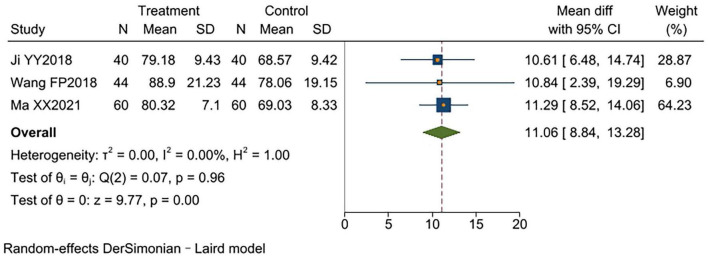
Forest plot of PaO_2._

Three studies (11, 14, 15) reported PaO2, involving a total of 288 patients (144 per group). No significant heterogeneity was observed between studies (*I*^2^ = 0%, *p* < 0.00001). Fixed-effect model analysis revealed significantly higher PaO2 levels in the experimental group compared to the control group [MD = 11.06, 95% CI (8.84, 13.28), *p* < 0.00001]. Results indicated that TCM-BDRT effectively elevated patients’ PaO2 levels (Figure 10).

#### 3.3.9 Incidence of adverse events

Two studies (18, 19) that monitored safety (such as complete blood count, urinalysis, stool analysis, liver and renal function, blood pressure, electrocardiogram, etc.) with a total of 128 patients (63 in the experimental group and 65 in the control group), no adverse events were reported, suggesting a favorable short-term safety profile for TCM-BDRT.

### 3.4 Publication bias assessment

**FIGURE 11:**
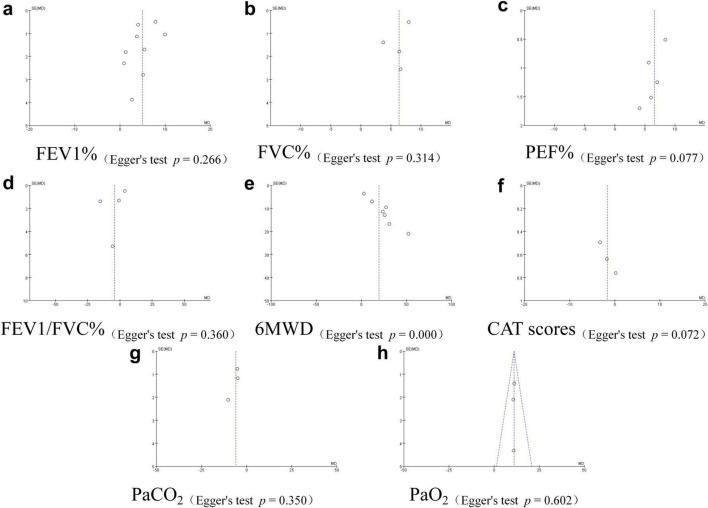
Publication bias funnel plot. Funnel plots **(a– h)** and Egger’s tests for each outcome measure. The results showed significant publication bias only for 6MWD (*p* = 0.000), while no significant bias was observed for the other measures (*p* < 0.05).

The funnel plot generated for the study examining pulmonary function indicators (FEV1%, FVC%, PEF%, FEV1/FVC%), exercise endurance (6MWD), CAT scores, and blood gas parameters (PaCO_2_, PaO2) revealed that, except for PaO2, which exhibited a broadly symmetric distribution, all other outcome measures displayed asymmetric scatter plots. As shown in Figure 11, this pattern strongly suggests publication bias. An Egger’s test was subsequently conducted, with all *p*-values except for 6MWD exceeding 0.05, indicating no significant publication bias. Finally, a “trim and fill” method was applied to estimate the adjusted combined effect size for publication bias. The adjusted effect size remained statistically significant (*p* < 0.01), consistent with the unadjusted findings. In conclusion, significant publication bias was present for 6MWD, but “trim and fill” method proved the findings were still robust (Figure 11 and the Supplementary material 5).

### 3.5 Certainty of evidence

The results indicate that the quality of evidence varies across different outcomes. The evidence for FEV1%, PEF%, and PaO_2_ was rated as moderate, suggesting that TCM-BDRT produced relatively consistent improvements in lung function and gas exchange, although the certainty was slightly downgraded due to heterogeneity and limited sample sizes. The evidence for FVC%, FEV1/FVC%, 6MWD, and PaCO_2_ was rated as low, as these outcomes were affected by high heterogeneity, limited overlap of confidence intervals, small sample sizes, or reporting bias. The CAT score was rated as very low, primarily due to study limitations, high heterogeneity, and insufficient sample sizes (Table 2).

**TABLE 2:** Certainty of evidence.

Certainty assessment							No. of patients		Effect	Quality	Important
No of studies	Design	Risk of bias	Inconsistency	Indirectness	Imprecision	Other considerations	[Intervent]	[Compare]	Effect size (95% CI)		
FEV1%											
9	RCTs	No serious	Serious^c^	No serious	No serious	None	404	406	MD = 5.00, 95% CI (2.95, 7.05)	⊕⊕⊕○ Moderate	CRITICAL
FVC%											
4	RCTs	No serious	Serious^c^	No serious	Serious^d^	None	171	171	MD = 6.34, 95% CI (4.04, 8.63)	⊕⊕○○ Low	CRITICAL
PEF%											
5	RCTs	No serious	Serious^c^	No serious	No serious	None	201	201	MD = 6.55, 95% CI (4.93, 8.18)	⊕⊕⊕○ Moderate	CRITICAL
FEV1/FVC%											
4	RCTs	No serious	Serious^c^	No serious	Serious^d^	None	185	185	MD = −4.28, 95% CI (−13.79, 5.24)	⊕⊕○○ Low	CRITICAL
6MWD											
7	RCTs	No serious	Serious^c^	No serious	No serious	Reporting bias^b^	305	307	MD = 19.53 m, 95% CI (7.94, 31.12)	⊕⊕○○ Low	CRITICAL
CAT Scores											
3	RCTs	Serious^a^	Serious^c^	No serious	Serious^d^	None	142	142	MD = −1.66, 95% CI (−3.62, 0.30)	⊕○○○ Very Low	IMPORTANT
PaCO2											
3	RCTs	No serious	Serious^c^	No serious	Serious^d^	None	144	144	MD = −5.99, 95% CI (−8.28, −5.99,	⊕⊕○○ Low	IMPORTANT
PaO2											
3	RCTs	No serious	No serious	No serious	Serious^d^	None	144	144	MD = 11.06, 95% CI (8.84, 13.28)	⊕⊕⊕○ Moderate	IMPORTANT

^a^Have not implemented allocation concealment and blinding methods.

^b^There is publication bias in this outcome.

^c^High heterogeneity and little overlapping confidence intervals.

^d^Sample size < 400.

## 4 Discussion

### 4.1 Overview of study findings

This study included 11 RCTs involving 964 COPD patients. Meta-analysis results demonstrated that TCM-BDRT significantly improved pulmonary function (FEV1%, FVC%, PEF%), exercise endurance (6MWD), and gas exchange function (decreased PaCO_2_, increased PaO2). This suggests that TCM-BDRT held significant value as a non-pharmacological intervention in the comprehensive management of COPD. However, no significant improvement in FEV1/FVC% or quality of life (CAT scores) was observed, indicating that the effects of this intervention may be targeted and limited in scope.

### 4.2 TCM perspective and clinical relevance of TCM-BDRT

TCM-BDRT may exert effects at multiple levels through a tripartite training model of “breath regulation, daoyin exercises, and mental focus” (20). Initially, slow, deep, and rhythmic breathing in TCM-BDRT reduces respiratory energy expenditure and enhances diaphragmatic contractility. It also improves thoracic compliance, collectively optimizing ventilation mechanics. These physiological improvements can increase key lung function parameters, such as FEV1% and PEF%, while also enhancing exercise tolerance and endurance. Furthermore, deep breathing promotes alveolar ventilation and improves the ventilation/perfusion ratio, thereby helping to lower PaCO_2_ levels and increase PaO2, which may alleviate symptoms of dyspnea and fatigue in COPD patients. Beyond mechanical effects, TCM-BDRT may facilitate more efficient gas exchange at the alveolar-capillary interface, supports oxygen uptake and carbon dioxide elimination during both rest and activity. Together, these mechanisms suggest that TCM-BDRT offers both functional and clinical benefits, and providing a feasible non-pharmacological adjunct to conventional COPD management by improving respiratory efficiency, exercise capacity, and overall quality of life. Psychological regulation was suggested to suppress sympathetic nervous system activation and alleviate anxiety, thereby indirectly reducing dyspnea and further improving exercise endurance (21). From a traditional Chinese medicine perspective, TCM-BDRT aligns with the theories that “the lung governs qi” and “the kidney is the root of qi,” embodying the holistic rehabilitation concept of “regulating the lung to promote qi circulation and fortifying the kidney to safeguard qi function” (22). This resonates with modern rehabilitation strategies that enhance qi function by tonifying the lung and kidney.

### 4.3 Heterogeneity and robustness of results

Moderate to high heterogeneity was observed in some outcome measures. Sensitivity analyses indicated that this heterogeneity primarily stemmed from studies that did not report disease durations or those with significant differences in control interventions (e.g., inspiratory muscle training or Western medication). Excluding these studies significantly reduced heterogeneity while maintaining or even increasing effect sizes. These findings suggest overall robustness in the effectiveness of TCM-BDRT interventions, yet underscore the importance of adopting more standardized approaches in patient stratification and control group design for future research.

### 4.4 Comparison with previous studies and guidelines

International guidelines recommend exercise training as the primary intervention for pulmonary rehabilitation in COPD (23). However, traditional exercise programs often face challenges such as low adherence and excessive exercise intensity, particularly for frail patients or those with limited exercise capacity. To our knowledge, this is the first systematic review focusing on TCM-BDRT for COPD rehabilitation, this study demonstrates that TCM-BDRT—characterized by low intensity, long-term feasibility, and cultural adaptability—can provide alternative or complementary treatment options for such patients. Their effects on improving lung function and exercise endurance align with previous small-sample studies, but this meta-analysis provides stronger evidence-based support. The lack of improvement in CAT scores may stem from factors such as insufficient intervention duration, limited sensitivity of assessment tools, and inadequate psychosocial support.

### 4.5 Strengths and limitations

This study’s strength lies in its comprehensive evaluation of TCM-BDRT on key indicators of COPD. It is the first review to include a large sample size, enhancing reliability. The findings demonstrate reliability and were validated through sensitivity analysis. TCM-BDRT exhibits characteristics of low intensity, high feasibility, and long-term sustainability, making it particularly suitable for patients with frailty or reduced exercise capacity, limited endurance, or difficulty engaging in conventional exercise. This intervention demonstrates scalability and adaptability across diverse cultural contexts. Furthermore, its mechanism of action aligns with both modern medical principles and TCM theory, facilitating integration into combined rehabilitation models that synthesize both therapeutic approaches.

However, this review has several limitations. Many included trials had small sample sizes and lacked adequate randomization and blinding, potentially introducing bias. Significant heterogeneity was observed due to differences in intervention duration (3–24 weeks), frequency (once or twice daily), and adjunct measures (health education, Western medication, standard care), resulting in moderate to high heterogeneity for some outcome measures. No statistically significant improvement in quality of life was observed, potentially attributable to short intervention periods, limited sensitivity of assessment tools, and inadequate psychosocial interventions. Furthermore, incomplete reporting of safety outcomes and lack of long-term follow-up data hindered comprehensive evaluation of the long-term efficacy and risks of TCM-BDRT.

### 4.6 Clinical implications and future research directions

TCM-BDRT may represent a promising non-pharmacological intervention for positively improving pulmonary function, enhancing exercise endurance, and optimizing gas exchange. This therapy is particularly beneficial for patient populations who struggle to adhere to or tolerate conventional exercise rehabilitation (24). With its low intensity, ease of implementation, TCM-BDRT offers a highly promising complementary approach to clinical pulmonary rehabilitation. Future research should focus on high-quality, multicenter, large-sample, long-term follow-up RCTs to determine optimal intervention duration, frequency, and intensity. Additionally, studies should explore synergistic effects between TCM-BDRT and conventional pulmonary rehabilitation, incorporating more patient-centered outcome measures (e.g., exacerbation rates, readmission rates, quality of life, and psychological status). Objective measures (e.g., diaphragm ultrasound, cardiopulmonary exercise testing, autonomic function assessment) should be employed to investigate the physiological mechanisms of TCM-BDRT, while safety should be evaluated using internationally standardized adverse event monitoring frameworks.

## 5 Conclusion

TCM-BDRT has demonstrated potential benefits in improving lung function, exercise endurance, and gas exchange capacity in patients with COPD, though additional clinical evidence is required to support its safety profile. As a low-intensity, scalable rehabilitation program integrating principles from both Chinese and Western Medicine, this therapy holds broad clinical application prospects. With the accumulation of high-quality evidence in the future, TCM-BDRT is expected to become a key component in the comprehensive rehabilitation management of COPD.

## Data Availability

The original contributions presented in the study are included in the article/Supplementary material, further inquiries can be directed to the corresponding author.
