# Supplementary material for: Effectiveness of traditional Chinese Medicine Breathing-Daoyin Rehabilitation Techniques on lung function and exercise endurance in COPD: a systematic review and meta-analysis
Source: Front Med (Lausanne). 2026 May 4;13:1775201. doi: 10.3389/fmed.2026.1775201 (PMC13180815; doi:10.3389/fmed.2026.1775201)
Supplement: Supplementary file 1 [file Table_1.docx]

Supplementary Materials

**Effectiveness of Traditional Chinese Medicine Breathing-Daoyin Rehabilitation Techniques on Lung Function and Exercise Capacity in COPD: A Systematic Review and Meta-Analysis**

# Supplementary Material 1 - Operation steps

| The specific steps of TCM Breathing-Daoyin Rehabilitation Techniques TCM Breathing-Daoyin Rehabilitation Techniques is a traditional Chinese medicine pulmonary rehabilitation technique developed by the Respiratory Team of the First Affiliated Hospital of Henan University of Chinese Medicine, with funding support from the National Traditional Chinese Medicine Industry Special Project (201107002). Developed under the guidance of the holistic TCM principles of "unity of body and spirit" and "harmony between human and nature," and based on theories such as yin-yang, the five elements, zang-fu organs, meridians, and essence-qi-spirit, this technique represents a novel approach to pulmonary rehabilitation. It has been granted copyright registration (National Copyright Registration No. 2013-A-00096834)[21]. | |
| --- | --- |
| **Section 1**: Commencing form to tranquilize the mind. Stand in a relaxed and tranquil state, relax the body, separate the feet to shoulder-width apart, close the eyes, touch the palate with the tongue, close the mouth/lips, draw in the chest and abdomen, lift the anus, naturally drop the arms, slightly bend the hip and knee joints, remove distracting thoughts and perform a 5-min abdominal breathing(**Figure 1**). | 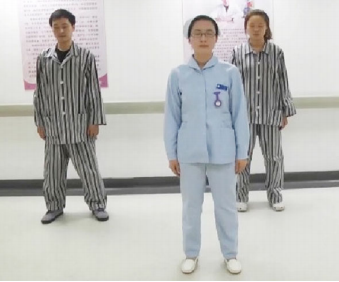 **Figure 1. Standing in a relaxed and tranquil state** |
| **Section 2**: Breathing to upper *Dantian* [around Yintang (GV 29)] and lower *Dantian* [around Guanyuan (CV 40)] to regulate lung qi. Stand with the feet together, move the left foot a step forward at an angle of 45° and lift the hands to upper *Dantian*. Slowly separate the hands and breathe in with the nose and breathe out with the mouth when closing the hands. Then, move hands and breathe in with the nose, and breathe out with the mouth when closing the hands. Repeat 3 times. Then repeat another 3 times with the right foot (**Figure 2**). | 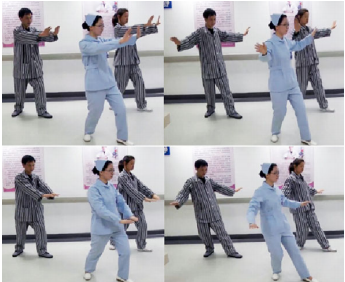 **Figure 2. Breathing to upper and lower *Dantian*** |
| **Section 3**: Regulating the lung and kidney. Slowly lift the arms from the side of the body with palms facing downward and turn the palms upward when the arms are fully extended. Slowly close the hands in front of the body and lift to the upper *Dantian*. Press down to the lower *Dantian*, bend over and flex the knee. Shift the body weight forward, gently touch the ground with Yongquan (KI 1), imagine a spring flowing from Yongquan (KI 1) to the sacrum along the medial aspect of the lower leg and thigh, and further ascend to pass through the kidney along the spine. Open the hands, allow the imagination to further ascend to pass through the diaphragm and enter the lung and then to the axillary fossa. Turn the palms upward and allow the imagination to reach Shaoshang (LU 11) along the Lung Meridian of Hand Taiyin (**Figure 3**). This section is the core part of breathing and *Daoyin* exercises, involving breathing, mental intent and body movements. This section is associated with the organs, the Lung Meridian and the Kidney Meridian. Since the kidney receives qi and is the root of qi and the lung dominates breathing, this exercise can regulate the depth of breathing and qi activities. | 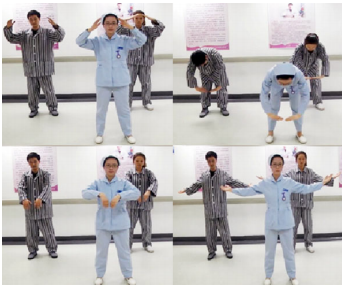 **Figure 3. Regulating the lung and kidney** |
| **Section 4**: Turning the body and extending the hands to both sides. Move the left foot one step to the left, gradually turn the upper body (90°), lift the hands to the waist and shift the body weight to the right leg. Then extend the hands backwards like a hawk spreading its wings and breathe in with the nose. Lift the hands to the level of the shoulders, extend the hands forward and downward and breathe out with an effort. Repeat 3 times. Then turn to the right side and repeat 3 times (**Figure 4**). This section aims to open the chest and assist breathing through combining body movements and abdominal breathing. | 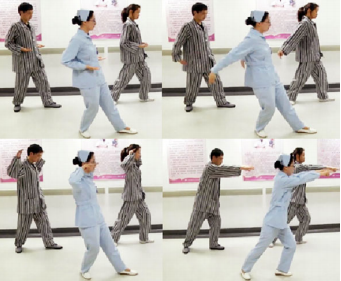 **Figure 4. Turning the body and extending the hands** |
| **Section 5**: Mo-rubbing Shenshu (BL 23) to warm kidney yang and strength the kidney’s function in receiving qi. Lift the hands from the side to Shenshu (BL 23) and rub 36 times using the great thenar and then return the hands to the lower abdomen along the side of the body (**Figure 5**). | 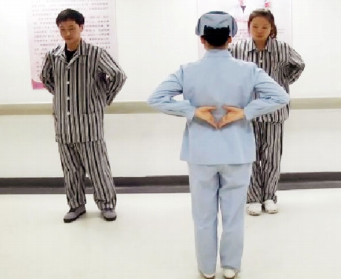 **Figure 5. Mo-rubbing Shenshu (BL 23)** |
| **Section 6**: Nourishing qi and concluding exercise. Place the overlapped hands on the lower abdomen, touch the palate with the tongue, tranquilize the mind (**Figure 6**). | 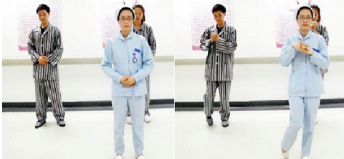 **Figure 6. Nourishing qi and concluding exercise** |
| Implementation method: The first month was considered intensive training, during which participants exercised for 30~60 minutes per session, twice daily, 5 times a week. They attended the hospital twice a week for centralized intensive training and guidance from a rehabilitation therapist, and exercise diaries were collected and distributed. On the remaining 3 days of the week, training was performed at home. After 4 weeks, participants entered an 8-week unsupervised training phase (each patient performed training according to the CD-ROM), exercising for 30~60 minutes per session, once or twice daily, at least 5 times a week. During this period, researchers conducted telephone follow-ups every 2 days, and participants visited the hospital once a month for on-site assessment and guidance [10-20, 21]. | |

# Supplementary Material 2 - search strategy

Search strategy for PubMed.（**2025-08-08**）

| Search query |
| --- |
| **#1** TCM Respiratory Daoyin and Rehabilitation Technology [Title/Abstract] OR TCM Respiratory Daoyin[Title/Abstract] OR Respiratory Daoyin[Title/Abstract] OR Breathing Daoyin[Title/Abstract]  **#2** Pulmonary Disease, Chronic Obstructive[MeSH Terms]  **#3** Pulmonary Disease, Chronic Obstructive[Title/Abstract] OR Chronic Obstructive Lung Disease[Title/Abstract] OR Chronic Obstructive Pulmonary Diseases[Title/Abstract] OR COAD[Title/Abstract] OR COPD[Title/Abstract] OR Chronic Obstructive Airway Disease[Title/Abstract] OR Chronic Airflow Obstructions[Title/Abstract] OR Chronic Airflow Obstruction[Title/Abstract]  **#4** random*[Title/Abstract]  **#5** #2 OR #3  **#6** #1 AND #4 AND #5 |
| **Results 1** |

**Table 2**

Search strategy for Embase.（**2025-08-08**）

| Search query |
| --- |
| **#1** 'TCM Respiratory Daoyin and Rehabilitation Technology':ab,ti OR 'TCM Respiratory Daoyin':ab,ti OR 'Respiratory Daoyin':ab,ti OR ' Breathing Daoyin ':ab,ti  **#2** 'Pulmonary Disease, Chronic Obstructive'/exp OR 'Pulmonary Disease, Chronic Obstructive':ab,ti OR 'Chronic Obstructive Lung Disease':ab,ti OR 'COAD':ab,ti OR 'COPD':ab,ti  **#3** #1 AND #2 |
| **Results 3** |

**Table 3**

Search strategy for WOS.（**2025-08-08**）

| Search query |
| --- |
| **#1** TS=("TCM Respiratory Daoyin and Rehabilitation Technology" or "TCM Respiratory Daoyin" or "Respiratory Daoyin" or " Breathing Daoyin ")  **#2** TS=("Pulmonary Disease, Chronic Obstructive" or "Chronic Obstructive Lung Disease" or "COAD"or "COPD")  **#3** #1 and #2 |
| **Results 0** |

**Table 4**

Search strategy for Cochrane.（**2025-08-08**）

| Search query |
| --- |
| **#1** (TCM Respiratory Doayin and Rehabilitation Technology):ti,ab,kw OR (TCM Respiratory Daoyin):ti,ab,kw OR (Respiratory Daoyin):ti,ab,kw OR (Breathing Daoyin):ti,ab,kw  **#2** MeSH descriptor: [Pulmonary Disease, Chronic Obstructive] explode all trees  **#3** (Pulmonary Disease, Chronic Obstructive):ti,ab,kw OR (Chronic Obstructive Lung Disease):ti,ab,kw OR (COPD):ti,ab,kw OR (COAD):ti,ab,kw  **#4** #2 OR #3  **#5** #1 AND #4 |
| **Results 0** |

**Table 5**

| Search strategy for CNKI. （**2025-08-08**） |
| --- |
| **#1** (SU=(中医呼吸导引康复技术) OR SU=(中医呼吸导引技术) OR SU=(中医呼吸导引) OR TKA=(中医呼吸导引康复技术) OR TKA=(中医呼吸导引技术) OR TKA=(中医呼吸导引))  **#2** (SU=(慢性阻塞性肺疾病) OR SU=(慢阻肺) OR SU=(慢性阻塞性呼吸道疾病) OR SU=(慢性阻塞性肺病) OR SU=慢性阻塞肺疾病) OR SU=(慢性气道阻塞) OR SU=(慢性气流阻塞) OR SU=(COPD) OR SU=(COAD) OR TKA=(慢性阻塞性肺疾病) OR TKA=(慢阻肺) OR TKA=(慢性阻塞性呼吸道疾病) OR TKA=(慢性阻塞性肺病) OR TKA=慢性阻塞肺疾病) OR TKA=(慢性气道阻塞) OR TKA=(慢性气流阻塞) OR TKA=(COPD) OR TKA=(COAD))  **#3** (SU=(随机) OR SU=(临床观察) OR SU=(RCT) OR SU=(临床研究) OR TKA=(随机) OR TKA=(临床观察) OR TKA=(RCT) OR TKA=(临床研究))  **#4** #1 AND #2 AND #3 |
| **Results 15** |

**Table 6**

| Search strategy for WANFANG. （**2025-08-08**） |
| --- |
| (主题:(中医呼吸导引康复技术 or 中医呼吸导引技术 or 中医呼吸导引)) and (主题:(慢性阻塞性肺疾病 or 慢阻肺 or 慢性阻塞性呼吸道疾病 or 慢性阻塞性肺病 or 慢性阻塞肺疾病 or 慢性气道阻塞 or 慢性气流阻塞 or COPD or COAD)) and (题名或关键词:(随机 or 临床观察 or RCT or 临床研究) or 摘要:(随机 or 临床观察 or RCT or 临床研究)) |
| **Results 25** |

**Table 7**

Search strategy for VIP. （**2025-08-08**）

| Search query |
| --- |
| ((M=(中医呼吸导引康复技术 OR 中医呼吸导引技术 OR 中医呼吸导引) OR R=(中医呼吸导引康复技术 OR 中医呼吸导引技术 OR 中医呼吸导引))) AND ((M=(慢性阻塞性肺疾病 OR 慢阻肺 OR 慢性阻塞性呼吸道疾病 OR 慢性阻塞性肺病 OR 慢性阻塞肺疾病 OR 慢性气道阻塞 OR 慢性气流阻塞 OR COPD OR COAD)) OR (R=(慢性阻塞性肺疾病 OR 慢阻肺 OR 慢性阻塞性呼吸道疾病 OR 慢性阻塞性肺病 OR 慢性阻塞肺疾病 OR 慢性气道阻塞 OR 慢性气流阻塞 OR COPD OR COAD))) AND (M=(随机 OR 临床观察 OR RCT OR 临床研究) OR R=(随机 OR 临床观察 OR RCT OR 临床研究)) |
| **Results 46** |

**Table 8**

Search strategy for CBM. （**2025-08-08**）

| Search query |
| --- |
| ("中医呼吸导引康复技术"[常用字段:智能] OR "中医呼吸导引技术"[常用字段:智能] OR "中医呼吸导引"[常用字段:智能]) AND ("慢性阻塞性肺疾病"[常用字段:智能] OR "慢阻肺"[常用字段:智能] OR "慢性阻塞性呼吸道疾病"[常用字段:智能] OR "慢性阻塞性肺病"[常用字段:智能] OR "慢性阻塞肺疾病"[常用字段:智能] OR "慢性气道阻塞"[常用字段:智能] OR "慢性气流阻塞"[常用字段:智能] OR "COPD"[常用字段:智能] OR "COAD"[常用字段:智能]) AND ("随机"[标题:智能] OR "临床观察"[标题:智能] OR "RCT"[标题:智能] OR "临床研究"[标题:智能]) |
| **Results 1** |

**Supplementary Material 3 - Subgroup analysis**

**1.FEV1%-subgroup analysis**

**
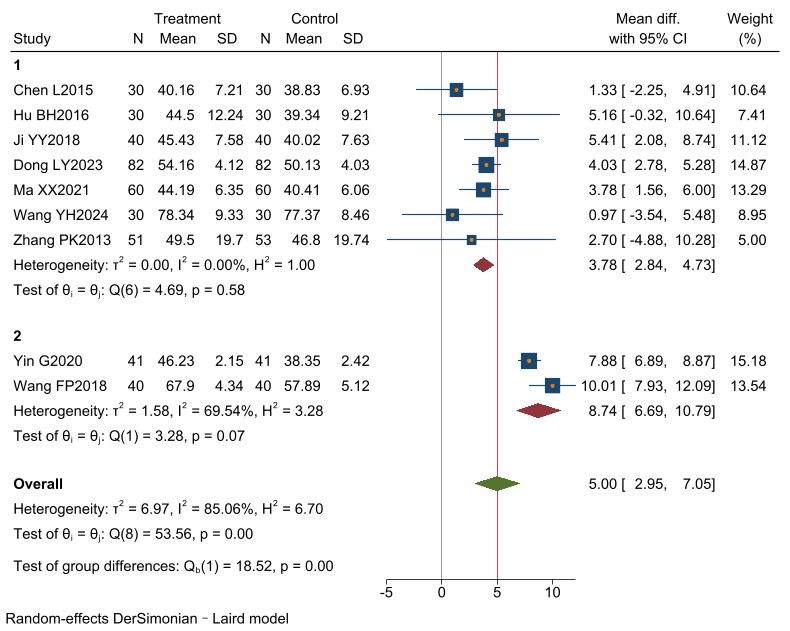
**

Note: 1:onset duration reported; 2:onset duration not reported

**2.FVC%-subgroup analysis**


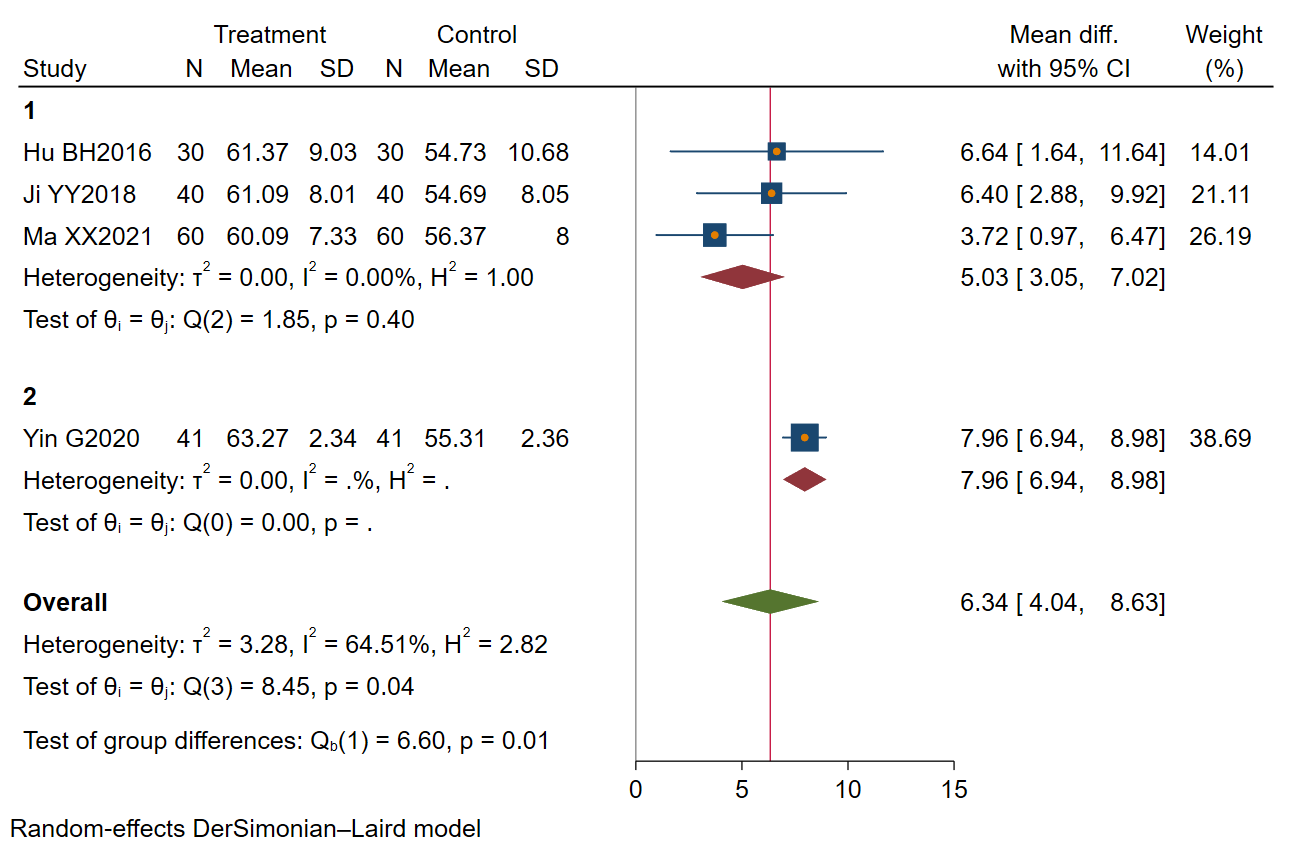


Note: 1:onset duration reported; 2:onset duration not reported

**3.PEF%-subgroup analysis**


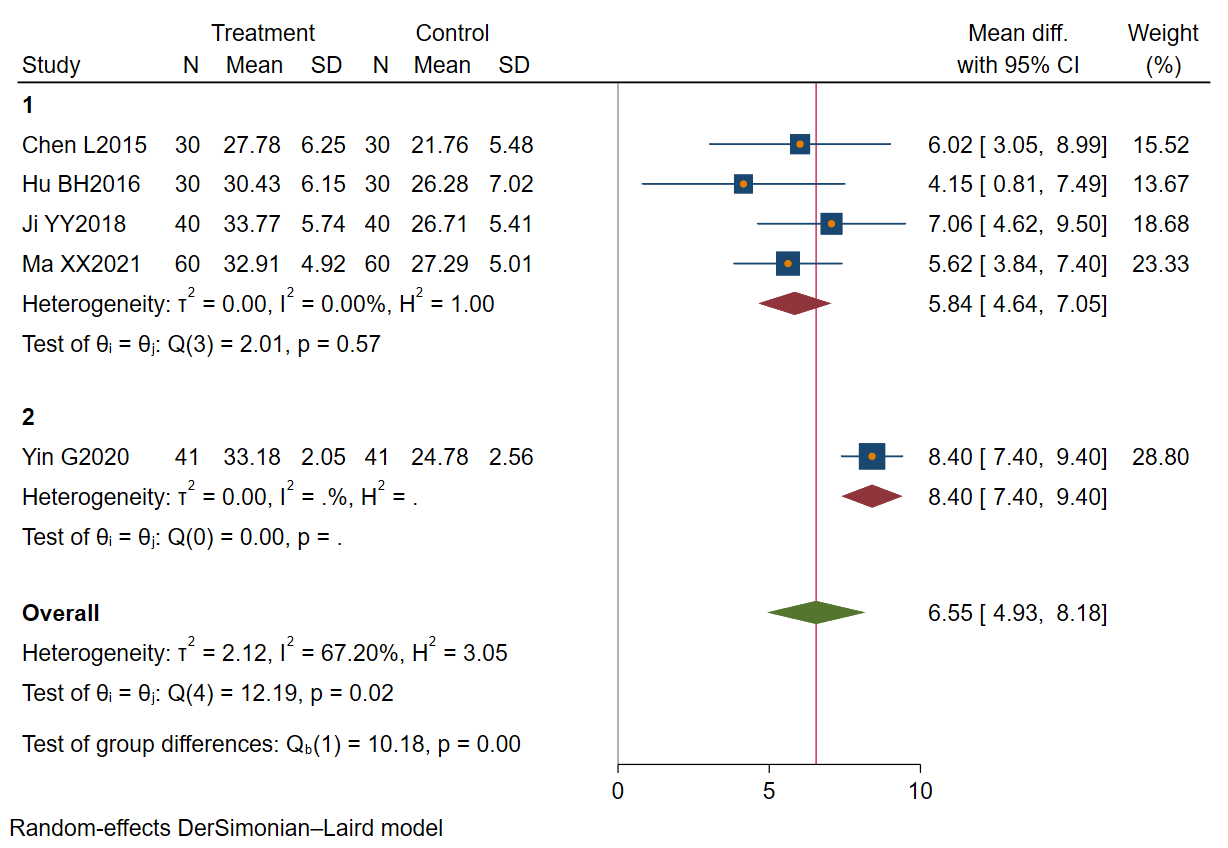


Note: 1:onset duration reported; 2:onset duration not reported

**4. 6MWD-subgroup analysis**

**
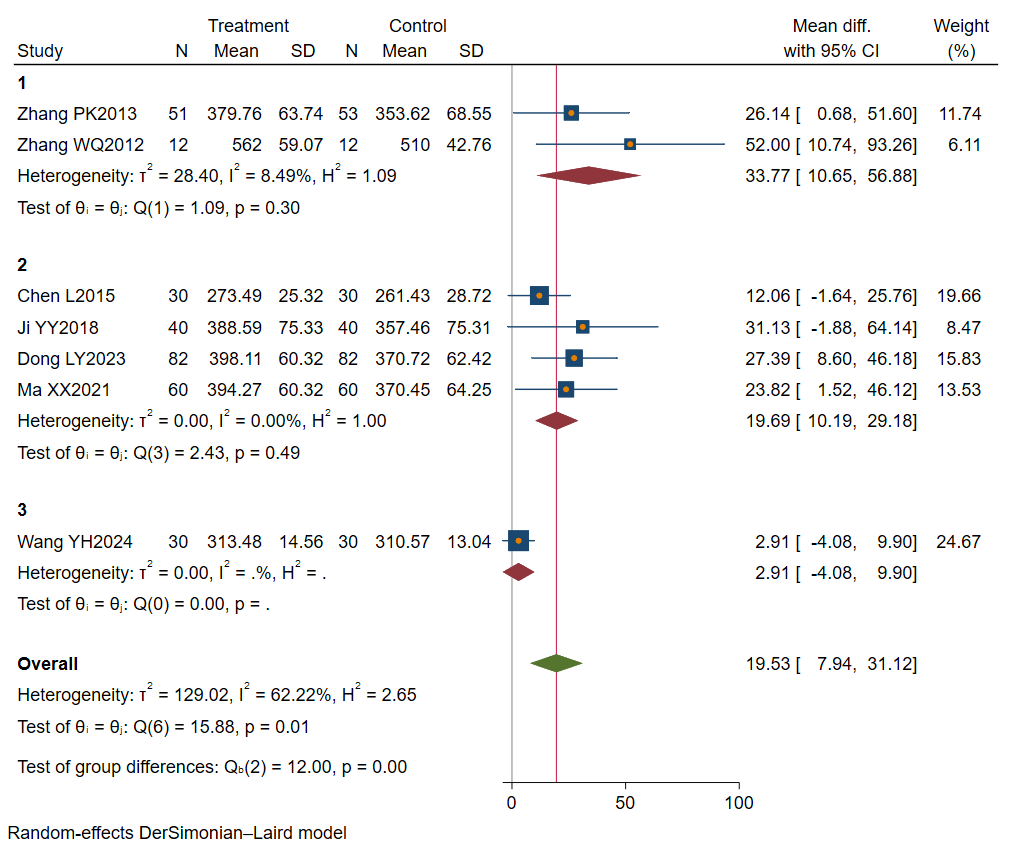
**

Note: 1:TCM-BDRT+HE *vs* HE; 2: TCM-BDRT+WM *vs* WM; 3: TCM-BDRT *vs* RMT

**5. PaCO_2_ -subgroup analysis**

#
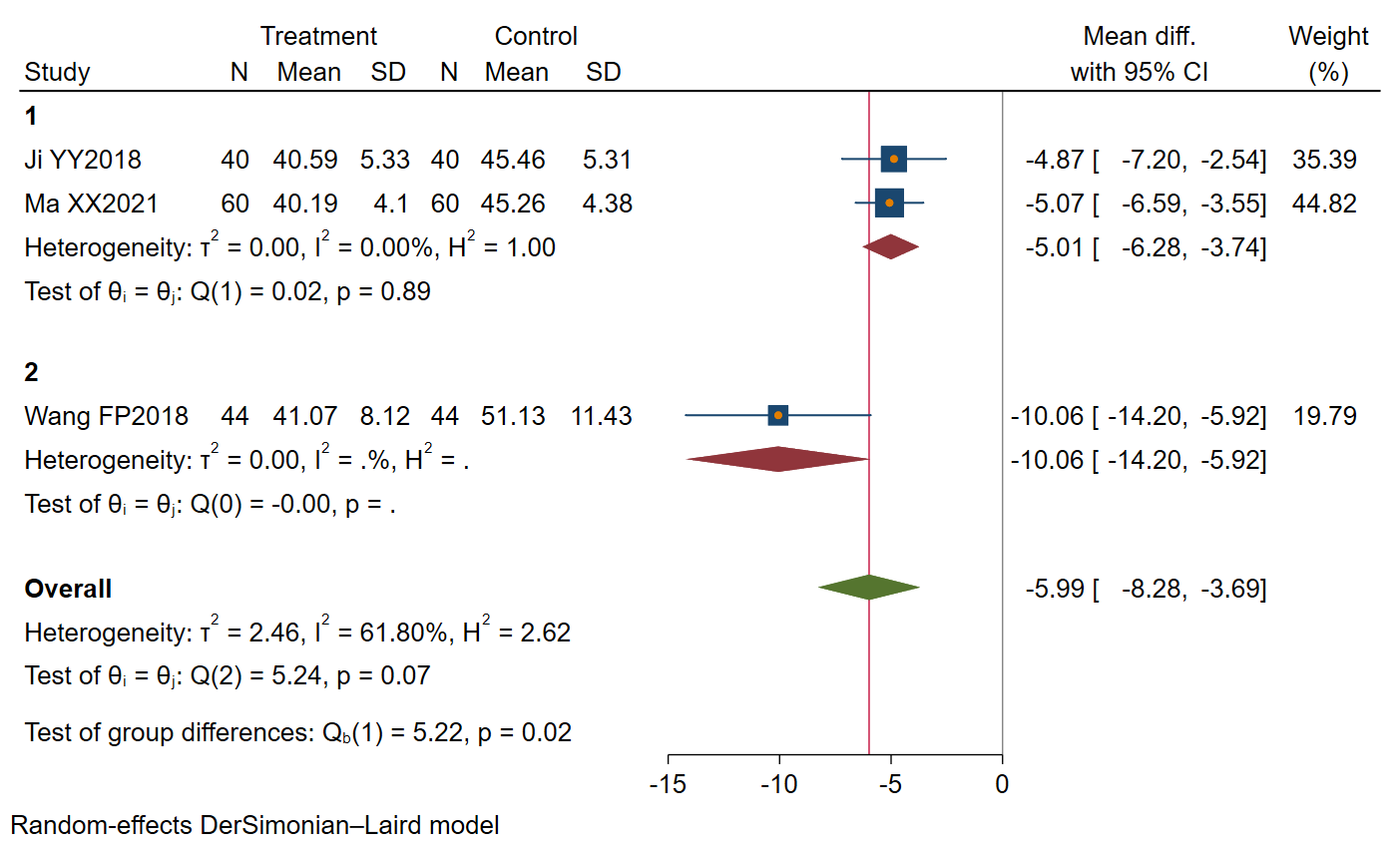


Note: 1:onset duration reported; 2:onset duration not reported

**Supplementary Material 4 - Leave-one-out sensitivity analysis**

1. **FEV1% - Leave-one-out sensitivity analysis**

1. **FVC% - Leave-one-out sensitivity analysis**

1. **PEF% - Leave-one-out sensitivity analysis**

1. **6MWD - Leave-one-out sensitivity analysis**

1. **PaCO_2_ - Leave-one-out sensitivity analysis**

# Supplementary Material 5 - Egger's test

**1. FEV1%-** **Egger's test**

metabias _meta_es _meta_se, egger

Note: data input format theta se_theta assumed

Egger's test for small-study effects:

Regress standard normal deviate of intervention

effect estimate against its standard error

.

Number of studies = 9 Root MSE = 2.516

------------------------------------------------------------------------------

Std_Eff | Coefficient Std. err. t P>|t| [95% conf. interval]

-------------+----------------------------------------------------------------

slope | 7.700112 1.523762 5.05 0.001 4.096988 11.30324

bias | -1.850766 1.530318 -1.21 0.266 -5.469392 1.767861

------------------------------------------------------------------------------

Test of H0: no small-study effects **P = 0.266**

**2. FVC%-** **Egger's test**

Egger's test for small-study effects:

Regress standard normal deviate of intervention

effect estimate against its standard error

.

Number of studies = 4 Root MSE = 1.495

------------------------------------------------------------------------------

Std_Eff | Coefficient Std. err. t P>|t| [95% conf. interval]

-------------+----------------------------------------------------------------

slope | 8.719277 1.235506 7.06 0.019 3.403323 14.03523

bias | -1.784449 1.337147 -1.33 0.314 -7.537729 3.968831

------------------------------------------------------------------------------

Test of H0: no small-study effects  **P = 0.314**

**3. PEF%-** **Egger's test**

Egger's test for small-study effects:

Regress standard normal deviate of intervention

effect estimate against its standard error

.

Number of studies = 5 Root MSE = 1.101

------------------------------------------------------------------------------

Std_Eff | Coefficient Std. err. t P>|t| [95% conf. interval]

-------------+----------------------------------------------------------------

slope | 9.713579 .9880567 9.83 0.002 6.569141 12.85802

bias | -2.981397 1.122235 -2.66 0.077 -6.55285 .5900563

------------------------------------------------------------------------------

Test of H0: no small-study effects **P = 0.077**

**4. FEV1/FVC%-** **Egger's test**

Egger's test for small-study effects:

Regress standard normal deviate of intervention

effect estimate against its standard error

.

Number of studies = 4 Root MSE = 7.073

------------------------------------------------------------------------------

Std_Eff | Coefficient Std. err. t P>|t| [95% conf. interval]

-------------+----------------------------------------------------------------

slope | 6.491978 5.361461 1.21 0.350 -16.57653 29.56048

bias | -7.126556 6.04936 -1.18 0.360 -33.15485 18.90174

------------------------------------------------------------------------------

Test of H0: no small-study effects  **P = 0.360**

**5. 6MWD-** **Egger's test**

Egger's test for small-study effects:

Regress standard normal deviate of intervention

effect estimate against its standard error

.

Number of studies = 7 Root MSE = .4381

------------------------------------------------------------------------------

Std_Eff | Coefficient Std. err. t P>|t| [95% conf. interval]

-------------+----------------------------------------------------------------

slope | -6.285475 2.246792 -2.80 0.038 -12.06104 -.5099129

bias | 2.692352 .3053677 8.82 0.000 1.90738 3.477325

------------------------------------------------------------------------------

Test of H0: no small-study effects **P = 0.000**

meta trimfill, estimator(linear) funnel

Effect-size label: Mean diff

Effect size: _meta_es

Std. err.: _meta_se

Nonparametric trim-and-fill analysis of publication bias

Linear estimator, imputing on the left

Iteration Number of studies = 9

Model: Random-effects observed = 7

Method: DerSimonian–Laird imputed = 2

Pooling

Model: Random-effects

Method: DerSimonian–Laird

---------------------------------------------------------------

Studies | Mean diff [95% conf. interval]

---------------------+-----------------------------------------

Observed |  **19.530** 7.940 31.119

Observed + Imputed | **15.841**  5.149 26.533

---------------------------------------------------------------

**6. CAT Scores-** **Egger's test**

Egger's test for small-study effects:

Regress standard normal deviate of intervention

effect estimate against its standard error

.

Number of studies = 3 Root MSE = .4418

------------------------------------------------------------------------------

Std_Eff | Coefficient Std. err. t P>|t| [95% conf. interval]

-------------+----------------------------------------------------------------

slope | -9.709295 .8768907 -11.07 0.057 -20.85125 1.432658

bias | 12.85767 1.455661 8.83 0.072 -5.638252 31.3536

------------------------------------------------------------------------------

Test of H0: no small-study effects **P = 0.072**

**7. PaCO_2_-** **Egger's test**

Egger's test for small-study effects:

Regress standard normal deviate of intervention

effect estimate against its standard error

.

Number of studies = 3 Root MSE = 1.197

------------------------------------------------------------------------------

Std_Eff | Coefficient Std. err. t P>|t| [95% conf. interval]

-------------+----------------------------------------------------------------

slope | -2.304836 2.065508 -1.12 0.465 -28.5496 23.93993

bias | -3.131812 1.921923 -1.63 0.350 -27.55216 21.28854

------------------------------------------------------------------------------

Test of H0: no small-study effects **P = 0.350**

**8. PaO_2_-** **Egger's test**

Egger's test for small-study effects:

Regress standard normal deviate of intervention

effect estimate against its standard error

.

Number of studies = 3 Root MSE = .2216

------------------------------------------------------------------------------

Std_Eff | Coefficient Std. err. t P>|t| [95% conf. interval]

-------------+----------------------------------------------------------------

slope | 11.50205 .6588987 17.46 0.036 3.129946 19.87415

bias | -.242312 .3359336 -0.72 0.602 -4.510753 4.026129

------------------------------------------------------------------------------

Test of H0: no small-study effects **P = 0.602**

Note: 1:onset duration reported; 2:onset duration not reported
